# Editorial: Rising stars in precision medicine 2021: imprecise medicine is unethical in the big data era

**DOI:** 10.3389/fmed.2023.1181788

**Published:** 2023-04-18

**Authors:** David S. Liebeskind

**Affiliations:** ^1^Neurovascular Imaging Research Core, University of California, Los Angeles, Los Angeles, CA, United States; ^2^Comprehensive Stroke Center and Department of Neurology, Geffen School of Medicine at the University of California, Los Angeles, Los Angeles, CA, United States

**Keywords:** precision medicine, data science, innovation, imaging, biomarkers, clinical trials

The publication of this open-access Research Topic on innovative approaches to and aspects of precision medicine across a diverse set of clinical conditions underscores the recent evolution and ethical considerations of translational research in medicine. The Research Topic includes 17 distinct articles on data science methods across a diverse range of conditions to discern the impact of baseline patient characteristics, medical decision-making and specific therapeutic interventions on outcomes (Chen J-Q. et al.; Chen X-M. et al.; Chen Y. et al.; Choudhury et al.; Deng et al.; Du et al.; Geng et al.; He et al.; Huang, Liu et al.; Huang, Zhang et al.; Ke et al.; Kong et al.; Li et al.; Li and Gong; Ma et al.; Yu et al.; Zhao et al.). A variety of biomarkers are used, including standard diagnostic tests, imaging, proteomics and genomics to classify and study subsets of disease with novel methodologies. The clinical topics vary from oncology to pulmonary disease, dermatology, and traumatic brain injury. A variety of patient-specific biomarkers are highlighted, including genetic profiles, RNA signatures and routine serum assays that may be used to more precisely classify individual patients and predict their long term outcomes or therapeutic response. The broad range of highly specialized conditions covered on this topic is expansive yet demonstrates the critical roles of open access publication in data sharing, translation of data science methods across disciplines, and real-world data to drive future improvements in patient outcomes.

Precision medicine is not new. Although references to precision medicine have proliferated since the Human Genome Project, focusing extensively on individual-specific genetic characteristics, the conceptual basis has been implemented in medical practice for centuries. Since ancient times, it has been understood that individual patients respond differently to the same treatments due to underlying biological diversity in the pathophysiology or patient-specific features. Genomics, however, did provide a great example of how biomarkers may be employed in translational research. Although monogenic disorders may occur, most disease states likely involve complex and potentially, subtle, genome-wide associations, and interactions with environmental factors during life. The genomics, proteomics and other biomarkers are likely informative of potential therapeutic response to specific interventions at different disease stages or timing.

The imprecise medicine of many traditional clinical research paradigms has skewed the development of novel therapeutics and has often failed to address the overwhelming disparities manifest in patient presentations around the world, as only select patient populations and standard analytic approaches have been applied. Paradoxically, clinical research methodologies have been ensconced in concerns regarding ethical principles, benevolence and doing what is right for each individual patient, yet perpetuating imprecise medicine is inherently, unethical. Informed consent of patients and institutional review of research protocols enhance ethics of specific studies or clinical trials, yet biases regarding healthcare disparities, access to care and access to research remain tremendous challenges. Numerous clinical and translational research vehicles exist, such as phased clinical trials with the ultimate randomized, controlled trial (RCT) as the pinnacle, yet multiple alternative pathways exist; however, they are less valued. RCTs are overly obsessed with data collection, with paradoxical paucity of details on data quality or validity, such as independent adjudication of imaging measures by a core lab or related methodology. Post-marketing surveillance of most therapeutic drugs or devices is largely non-existent in most geographies around the world, including the United States. Registries and vehicles to evaluate quality of healthcare delivery are largely unfunded, unregulated, and devoid of validated data checks or measures to assess generalizability in routine clinical practice across most geographical regions.

Perhaps such gaps in translational research could be excused in prior years, yet the abundance of patient-specific information in the current big data era should prompt a reconsideration of traditional research paradigms. The big data era has not only sparked interest in large, diverse datasets, as it has also heralded the emergence of big data analytics, including artificial intelligence methods that can be readily deployed with current clinical data. In the United States, The Health Insurance Portability and Accountability Act of 1996 (HIPAA) was intended to promote data sharing and interoperability of clinical data systems, rather than to solely ensure individual patient privacy. Unfortunately, HIPAA has been cited to restrict data sharing rather than the original purpose of portability and dissemination of data and related findings. Data sharing at the National Institutes of Health and other platforms is a now a top priority. In routine care, the electronic health record systems, individual hospitals, institutions, provider groups, and academicians limit data sharing due to competition with others and the relative strength in controlling or restricting such data access. The marked expansion of knowledge with artificial intelligence is almost unbelievable with recent advances such as ChatGPT and likely future development of related medical applications, yet access to data is a starting point. Medical or clinical data has already been simplified and codified by common data elements or variables that exist in most medical specialties, laboratory assays and medical imaging modalities. Simultaneously, data scientists know of such potential yet lack the clinical expertise to apply such analytical techniques to specialized clinical topics. Unsupervised machine learning or artificial intelligence will likely always depend on periodic retraining by expert annotations or clinical guidance, yet patterns may rapidly emerge from machine learning that human efforts would take generations to realize. Unlike recent concerns over false ground truths inadvertently used to train machine learning, most medical data relate to timed, quantitative measures of biological significance such as physiological measures (e.g., vital signs), laboratory values, imaging, and increasing use of functional outcome measures that are widely available.

These changes sparked by the big data era and continued practice of imprecise medicine pose an ethical conundrum regarding current clinical research methodology and the responsibility of various parties. Academicians often initiate, design and perpetuate clinical research constructs such as trial design, the nature of clinical trials, statistical measures employed, and many entrenched traditional approaches, yet there is no concrete imperative to innovate such methodology. Industry partners are often seen as biased in trying to accelerate innovation of products, yet they need clinical research data not just leading up to RCTs, but afterwards in routine clinical practice. Regulatory bodies revert to predicate methods without clear imperative to facilitate advances, with a much larger concern for patent safety, far before efficacy. Yet once approved, most therapies go largely unmonitored in the general population. Fortunately, regulatory bodies are now implementing diversity requirements in prospective clinical trials. Routine healthcare providers remain relatively passive, as they can only use established methods or approved products. The most responsible and ethical approach for all would start with dissemination of data to collaboratively mine and discern potentially subtle yet pivotal insights on how precision medicine of individual patient response to a specific therapy is practiced on a daily basis around the world.

Open access publications, as in this Research Topic, and collaborative data sharing are critical in the big data era and further advances in precision medicine. This selective example of research articles provides an example to demonstrate such progress toward a broader swath of specific medical disciplines or specialties to be covered in future years. There is undoubtedly an indirect yield or benefit of disruptive innovation that may emerge from applying such novel methodologies or even medical therapies such as drugs or devices to other medical conditions. Open access is a critical barrier to overcome disparities in low income countries, to encourage collaboration and to promote data sharing as the initial steps in our transformation from imprecise medicine to precision medicine of the 21^st^ century.

## Author contributions

DL conceived the design and content of this editorial manuscript, drafted, revised, and approved the submitted version.

